# Bone marrow CD34^+^ cell subset under induction of moderate stiffness of extracellular matrix after myocardial infarction facilitated endothelial lineage commitment in vitro

**DOI:** 10.1186/s13287-017-0732-x

**Published:** 2017-12-13

**Authors:** Shuning Zhang, Xin Ma, Junjie Guo, Kang Yao, Cong Wang, Zhen Dong, Hong Zhu, Fan Fan, Zheyong Huang, Xiangdong Yang, Juying Qian, Yunzeng Zou, Aijun Sun, Junbo Ge

**Affiliations:** 1 Department of Cardiology, Zhongshan Hospital, Fudan University, Shanghai, China; 2 Institute of Cardiovascular Diseases, Fudan University, Shanghai, China; 3 Shanghai Cardiovascular Medical Center, Fudan University, Shanghai, China; 4 Institutes of Biomedical Sciences, Fudan University, Shanghai, China; 5 Institute of Pan-vascular Medicine, Fudan University, Shanghai, China; 6 Department of Cardiology, The Affiliated Hospital of Qingdao University, Qingdao University, Shandong, China

**Keywords:** Myocardial ECM stiffness, Myocardial infarction, CD34, Endothelial

## Abstract

**Background:**

The stiffness of the myocardial extracellular matrix (ECM) and the transplanted cell type are vitally important in promoting angiogenesis. However, the combined effect of the two factors remains uncertain. The purpose of this study is to investigate in vitro the combined effect of myocardial ECM stiffness postinfarction with a bone marrow-derived cell subset expressing or not expressing CD34 on endothelial lineage commitment.

**Methods:**

Myocardial stiffness of the infarct zone was determined in mice at 1 h, 24 h, 7 days, 14 days, and 28 days after coronary artery ligation. Polyacrylamide (PA) gel substrates of different stiffnesses were prepared to mechanically mimic the myocardial ECM after infarction. Mouse bone marrow-derived CD34^+^ and CD34^–^ cells were seeded on the flexible PA gels. The double-positive expression for DiI-acetylated low-density lipoprotein (acLDL) uptake and fluorescein isothiocyanate-*Ulex europaeus* agglutinin-1 (FITC-UEA-1) binding, the endothelial lineage antigens CD31, von Willebrand factor (vWF), Flk-1, and VE-cadherin, as well as cytoskeleton were measured by immunofluorescent staining on day 7. Cell apoptosis was evaluated by both immunofluorescent staining and flow cytometry at 24 h after culture.

**Results:**

We found that the numbers of the CD34^+^ cell subset adherent to the flexible substrates (4–72 kPa) was much larger than that of the CD34^–^ subset. More double-positive cells for DiI-acLDL uptake/FITC-UEA-1 binding were seen on the 42-kPa (moderately stiff) substrate, corresponding to the stiffness of myocardial ECM at 7–14 days postinfarction, compared with those on substrates of other stiffnesses. Similarly, the moderately stiff substrate showed benefits in promoting the positive expressions of the endothelial lineage markers CD31, vWF, Flk-1, and VE-cadherin. In addition, the cytoskeleton F-actin network within CD34^+^ cells was organized more significantly at the leading edge of the adherent cells on the moderately stiff (42 kPa) or stiff (72 kPa) substrates as compared with those on the soft (4 kPa and 15 kPa) substrates. Moreover, the moderately stiff or stiff substrates showed a lower percentage of cell apoptosis than the soft substrates.

**Conclusions:**

Infarcted myocardium-like ECM of moderate stiffness (42 kPa) more beneficially regulated the endothelial lineage commitment of a bone marrow-derived CD34^+^ subset. Thus, the combination of a CD34^+^ subset with a “suitable” ECM stiffness might be an optimized strategy for cell-based cardiac repair.

**Electronic supplementary material:**

The online version of this article (doi:10.1186/s13287-017-0732-x) contains supplementary material, which is available to authorized users.

## Background

A great deal of attention has been given to optimizing the cell treatment approach for myocardial infarction (MI) [1, 2]. The commitment of engrafted cells into vascular endothelial cells within damaged myocardium is regarded as one of the major methods for promoting cell-based cardiac repair [3]. The myocardial tissue microenvironment in the injured area and the type of transplanted cells are important factors in promoting stem cell specification in the infarct zone [4]. The physical properties of tissue extracellular matrix (ECM), such as stiffness, regulate stem cell adhesion, proliferation, migration, differentiation, and fate [5–7]. After MI, cardiac tissue stiffness changes from flexible to rigid in a time-dependent manner [8]. The consecutive changes in myocardial ECM stiffness might result in the differences in the survival of engrafted cells and cell lineage commitment, which are expected to determine the cell therapeutic effect. Thus, the stiffness of the myocardium ECM might determine which time point is optimal for cell repair for the infarcted myocardium, and the matrix property might also affect stem cell specification capacity differently for the type of stem cell. Bone marrow-derived mononuclear cells (BMMNCs) are the most commonly used cell lineage in clinical studies. Our previous study showed that the optimal time frame for implantation of BMMNCs after MI was1 to 2 weeks after the infarction [9]. Myocardial ECM stiffness within this time domain (elastic modulus ~ 42kPa) was more suitable for BMMNCs to differentiate into endothelial lineage cells and commit to angiogenesis. These favorable effects subsequently transferred to improved left ventricular systolic function and enhanced remodeling [9]. However, BMMNCs represented an unselected and mixed cell population. It is important to further determine whether matrix stiffness could selectively affect the BMMNC cell subsets and optimal conditions for the endothelial lineage commitment. A bone marrow-derived CD34^+^ cell subset, a well characterized population of stem cells, are powerful endothelial progenitor cells, and transplantation of this cell subset significantly enhances the formation of new blood vessels in injured hearts [10, 11]. However, it is not clear whether the CD34^+^ subset also presents the same ECM stiffness-dependent differentiation principle as BMMNCs. In addition, it is not known whether the cell lineage commitment is different between CD34^+^ and CD34^–^ cell subsets cultured on substrates with different stiffnesses. In the present study, we simulated myocardial ECM stiffness at different time points after infarction using an in vitro system, and investigated the effect of matrix stiffness as well as the expression of the cell surface marker CD34 of BMMNCs on endothelial lineage commitment.

## Methods

### Isolation of mouse BMMNCs

Femurs from 6-week-old male Balb/c mice were flushed three times in phosphate-buffered saline (PBS) with a 26-G needle to collect bone marrow cells. BMMNCs were isolated by density gradient centrifugation using Ficoll-Paque separator liquid (1.083 g/ml; Sigma-Aldrich, USA) using an established protocol [12]. Briefly, 3 μl of cell suspension was carefully laid over 3 ml of Ficoll-Paque liquid in a 15-ml conical tube, and centrifuged 2000 rounds/min for 30 min at 4 °C. The mononuclear cell layer at the interphase was transferred to a new 15-ml conical tube, washed twice, and then resuspended in complete M199 culture medium (Gibco, USA). Flow cytometry was performed to determine the percentage of CD34^+^and CD34^–^ subsets among BMMNCs.

### Isolation of CD34^+^ and CD34^–^ cell subsets

CD34^+^ and CD34^–^ subsets were isolated from mouse BMMNCs using a magnetic activated cell sorting (MACS) system. The collected BMMNCs were sensitized with FITC-labeled rat anti-mouse CD34 monoclonal antibodies (BD Biosciences, USA), and then incubated with anti-FITC microbeads (Miltenyi Biotec, Germany). The cells labeled with anti-CD34 antibodies and microbeads were enriched by MACS separator and MS columns, and the CD34^+^ cells were then released from the Dynabeads. To increase the purity of the CD34^+^ cells, we re-separated the first collected cell fraction with a second MS column. The remaining unlabeled CD34^–^ cell subset was also collected as the control. The purity of the collected CD34^+^ cell subset was detected by flow cytometry (BD Biosciences, USA), and the viability was assessed using methylene blue before cell culture.

### Determination of myocardial ECM stiffness postinfarction

Fifteen mouse models of MI were prepared by the ligation of the left anterior descending coronary artery in 6-week-old male Balb/c mice. The stiffness (elastic modulus, *E*) of the infarcted myocardial ECM at 1 h, 24 h, 7 days,14 days, and 28 days after MI were measured by atomic force microscopy, as described in our previous study [9]. Briefly, the fresh hearts post-MI were dissected and stored in 0.9% sodium chloride solution (*n* = 4 per time point). Hearts were then sectioned parallel to the longitudinal axis of the left ventricle from the vascular ligation point to the cardiac apex to yield tissue samples ~ 0.5 mm in thickness. Heart samples, which were mounted and immobilized on coverglass with adhesive tape, were placed on an atomic force microscope (Nanoscope IIIa, USA), and indented by an apyramid-tipped cantilever with spring constant of 60 pN/nm (Nanoprobes, USA) using a contact mode in 0.9% sodium chloride solution at room temperature. Mechanical information at 10 positions per sample was obtained and at each position five force-indentation plots were recorded. NanoScope software 5.30 (Veeco, USA) was used to acquire images. Elastic modulus was calculated based on the formula previously described by Domke [13] (1 h after MI: *E* = 17.94 ± 0.39 kPa; 24 h: *E* = 4.21 ± 0.16 kPa; 7 days: *E* = 31.38 ± 0.75kPa; 14 days: *E* = 53.23 ± 0.75 kPa; 28 days: *E* = 90.22 ± 2.97 kPa).

### In vitro simulation of myocardial ECM stiffness using the matrix gel system

Flexible polyacrylamide (PA) gel substrates were used to mimic the myocardial ECM stiffness at four time points postinfarction (1 h, 24 h, days 7–14 and days 14–28 post-MI). Briefly, 0.1 N NaOH was poured onto a ~ 20-mm round glass as a cleaning process (part of a 35-mm culture dish; Shengyou, China). 3-aminopropyltri-methoxysilane (Sigma-Aldrich, USA) was spread evenly onto the bottom and then washed with distilled H_2_O; 0.5% glutaraldehyde (Sigma-Aldrich, USA) was then used to increase the binding ability of the PA gels to glass substrates. Thereafter, the PA gel solution was prepared with various volume ratios of 2% bisacrylamide to 40% acrylamide. To induce bisacrylamide crosslinking, 10% ammonium persulphate and N,N,N,N-tetramethylethylenediamine were added to the PA gel solutions. Drops of PA gel solution were subsequently added to the glutaraldehyde-treated aminosilanized round glass, and chlorosilanized round coverslips (~18 mm) were placed on top of the PA solution. The substrates polymerized between the round coverslip and the glass bottom as thin films. When the substrates were polymerized for 30 min, the coverslips were carefully removed and the gels were washed with 50 mM HEPES three times. The gels were then activated by using a heterobifunctional agent, sulfo-SANPAH (Thermo Fisher Scientific, USA), and chemically crosslinked with fibronectin (10 mg/ml; Biosource, USA). The fibronectin coated on the four flexible substrates was labeled using mouse anti-human fibronectin antibody (R&D Systems, USA) and Alex Fluo 488-labeled goat anti-mouse secondary antibody (Invitrogen, USA), and was detected by the immunofluorescence method. No significant differences were seen in the concentrations of the coated fibronectin among the flexible substrates (Additional file 1). Four PA gel substrates with stiffnesses of 4 kPa, 15kPa, 42 kPa, and 72 kPa mechanically mimicked the myocardial ECM at 24 h, 1 h, days 7–14, and days 14–28 after MI, respectively [9]. Before being used for cell culture, the culture substrates were washed with PBS and exposed to UV light with PBS immersion for 15 min for sterilization purposes. Thereafter, the PBS was removed and replaced with Medium199 (25 mM HEPEs; Sigma) to allow equilibrium by putting the dishes in an incubator for at least 2 h.

### Cell culture on the flexible substrates

**Fig. 1:**
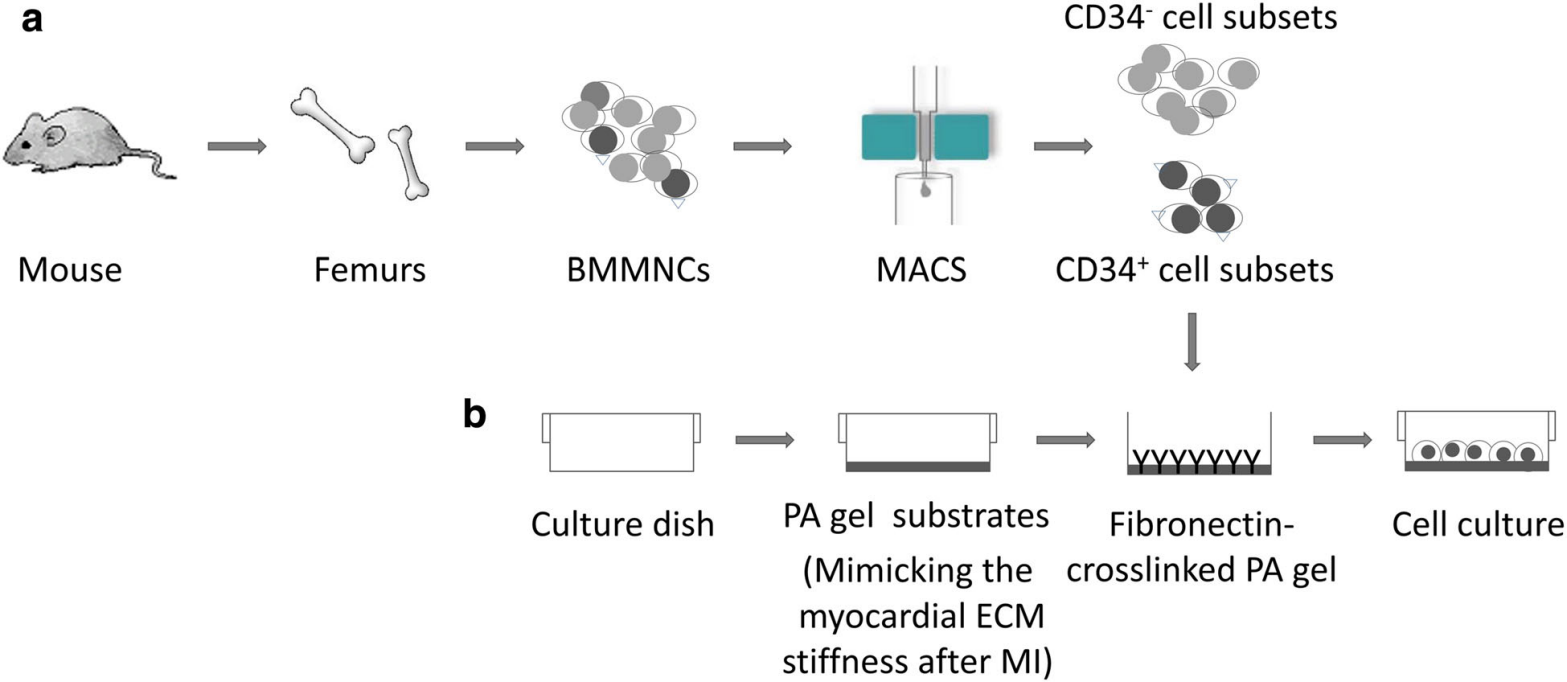
Study flowchart. **a** CD34^+^/CD34^–^ cell isolation; **b** flexible substrate preparation and cell culture. *BMMNCs* bone marrow-derived mononuclear cells, *ECM* extracellular matrix, *MACS* magnetic activated cell sorting, *MI* myocardial infarction, *PA* polyacrylamide

Mouse CD34^+^ and CD34^–^ cell subsets isolated from BMMNCs were suspended in complete Medium199 (Sigma) containing 20% fetal bovine serum (FBS) and 2.5 ng/ml vascular endothelial growth factor (VEGF; Peprotech, USA). These two cell subsets were seeded at a density of 5 × 10^5^ cells/dish in the above prepared culture dishes, respectively. The dishes were maintained at 37 °C in an incubator containing 5% CO_2_. After 48 h in culture, nonadherent cells were removed and adherent cells were cultured continuously. Culture medium was changed every 48 h. A flowchart of CD34^+^ and CD34^–^ subset isolation, the flexible substrate preparation, and the cell culture conditions are shown in Fig. 1.

### Acetylated low-density lipoprotein (acLDL) uptake and *Ulex europaeus* agglutinin-1 (UEA-1) binding test

Endothelial progenitor cells were characterized as the adherent cells double-positive for DiI-acLDL (Biomedical Technologies, USA) uptake and fluorescein isothiocyanate (FITC)-UEA-1 (Sigma, USA) binding. After 7 days in culture, the adherent cells were incubated with 10 μg/ml DiI-acLDL for 12 h at 37 °C, fixed with 4% paraformaldehyde (Sigma-Aldrich, USA), and then stained with 10 μg/ml FITC-UEA-1 for 3 h at room temperature. Nuclei of the cells were counterstained with 1 μg/ml 4',6-diamidino-2-phenylindole (DAPI; Roche, USA) for 15 min at room temperature. The cells were then visualized at 200× magnification with a laser scanning confocal microscope (LSM710; Carl Zeiss, Germany).

### Identification of surface markers of endothelial lineage cells

The adherent cells were rinsed with PBS and fixed in 4% paraformaldehyde for 15 min at room temperature. The cells were incubated in normal goat serum (3 mg/ml; Jackson ImmunoResearch, USA) for 20 min and then incubated with primary antibodies overnight at 4 °C. The cells were washed with PBS four times and then incubated with the corresponding secondary antibodies for 1.5 h. The primary antibodies included the purified rat anti-mouse CD31 (1:10, BD Biosciences, USA), vWF (1:100, Santa Cruz Biotechnology, USA), Flk-1 (1:100, Santa Cruz Biotechnology, USA), and VE-Cadherin (1:100, Santa Cruz Biotechnology, USA). The second antibodies were Alexa Fluor 594 chicken anti-rat IgG (H&L) and Alexa Fluor 488 chicken anti-rabbit IgG (H&L) (1:200, Invitrogen, USA). Nuclei were counterstained with 1 μg/ml DAPI (Roche, USA). Fluorescent images were visualized at 200× magnification with a laser scanning confocal microscope.

### Cytoskeletal staining

After being fixed in 4% paraformaldehyde, the adherent cells were stained overnight at 4 °C with anti-paxillin antibody (Abcam, USA) diluted at 1:100 in PBS buffer (0.02% NaN_3_, 3% bovine serum albumin (BSA) and 0.2% Triton X-100). Subsequently the labeled cells were stained with goat anti-rabbit IgG (H&L) antibody (Abcam, USA) diluted at 1:200 in PBS buffer (0.02% NaN_3_, 3% BSA) at room temperature for 1.5 h. The cells were then incubated at room temperature with phalloidin-TRITC (Sigma-Aldrich, USA) diluted 1:1000 in PBS buffer (0.1% Triton X-100). Finally, nuclei were stained with 1 μg/ml DAPI for 15 min at room temperature. It was difficult for adhesive cells to be trypsinized from the flexible substrates at day 7; due to the absence of sufficient cells, the present study did not carry out the initial study protocol on the semi-quantitative measurement of integrins and transmembrane receptors regulating cell-ECM adhesion using Western blot. Cytoskeletons were observed at 200× and 630× magnification by a laser scanning confocal microscope. In addition, to elucidate the differences in cell morphology and extension on the flexible substrates, the areas and circumferences of adhesive cells were measured and calculated based on cell imaging at day 1 and day 7 using Graphpad Prism 5.0 software (GraphPad Software, USA).

### Detection of cell apoptosis and cell survival in CD34^+^ cells

After being cultured for 24 h, CD34^+^ cells were harvested by trypsinization and were subsequently stained using Annexin V-PE/7-AAD to evaluate cell apoptosis with an Apoptosis Detection Kit (BD Biosciences, USA). Apoptotic cells (Annexin V^+^/7-AAD^–^) were analyzed by FACScan (BD Biosciences, USA). The percentages of apoptotic cells were analyzed using FlowJo software (TreeStar, USA). Meanwhile, the adherent CD34^+^ cells were also processed with live/dead staining using the LIVE/DEAD Cell Imaging Kit (488/570) (Invitrogen, USA) at 24 h after culture. In brief, cells were co-stained with 2× stock premixed with Live Green vial and Dead Red vial for 15 min at room temperature. After being washed with PBS, the LIVE/DEAD cells were visualized at 200× magnification with a laser scanning confocal microscope.

### Statistical analyses

Five randomly selected microscopic fields per cell sample were collected and the number of positive cells per high-power field (HPF) was recorded. To increase the reproducibility of the measurements, three separate tests were conducted for each specific assay. Data are presented as means ± standard deviation. The statistical differences between ECM stiffnesses were tested using two-way analysis of variance (ANOVA). The differences between two cell subgroups were analyzed using Student's *t* test. *P* < 0.05 was considered as a statistically significant difference.

## Results

### ECM stiffness regulated the presentation of endothelial progenitor characteristics as DiI-acLDL uptake/FITC-UEA-1 binding

**Fig. 2:**
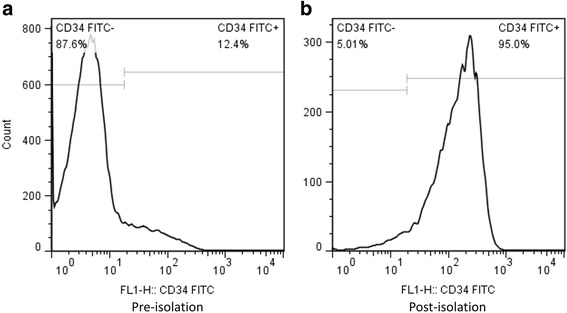
Flow cytometry for the percentage of CD34^+^ cells among BMMNCs (**a**) and the purity of isolated CD34^+^ cells (**b**). *FITC* fluorescein isothiocyanate

**Fig. 3:**
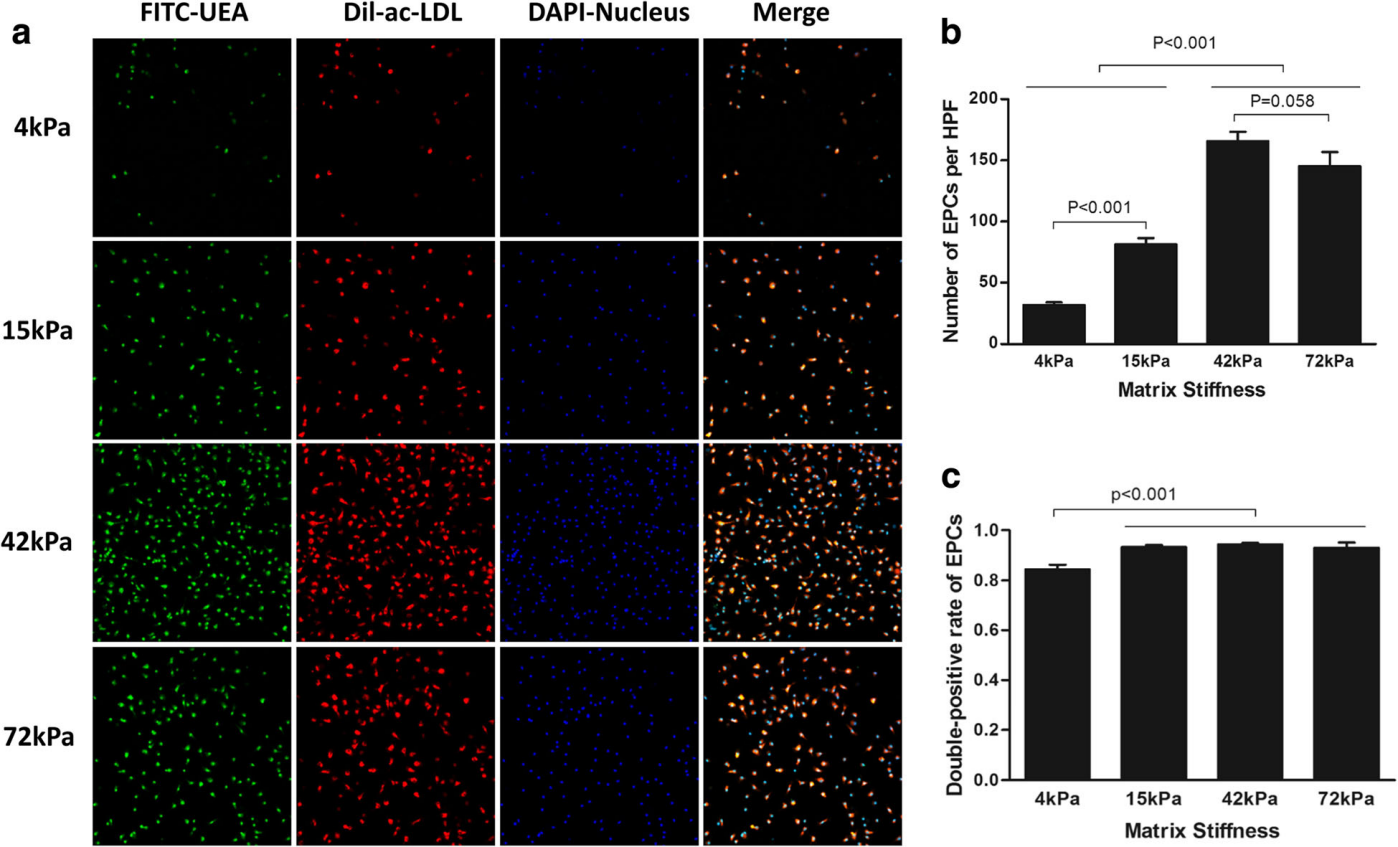
Double-positive cells for DiI-acLDL uptake and FITC-UEA-1 binding in CD34^+^ cell culture system with four different degrees of substrate stiffness. **a** Direct fluorescent staining; **b** comparisons of numbers of double-positive cells per HPF; **c** comparisons of rates of double-positive cells. Results are shown as mean + SD. *acLDL* acetylated low-density lipoprotein, *EPCs* endothelial progenitor cells, *HPF* high-power field, *acLDL* acetylated low-density lipoprotein, *DAPI* 4',6-diamidino-2-phenylindole, *FITC-UEA* fluorescein isothiocyanate-labeled *Ulex europaeus* agglutinin I lectin

**Fig. 4:**
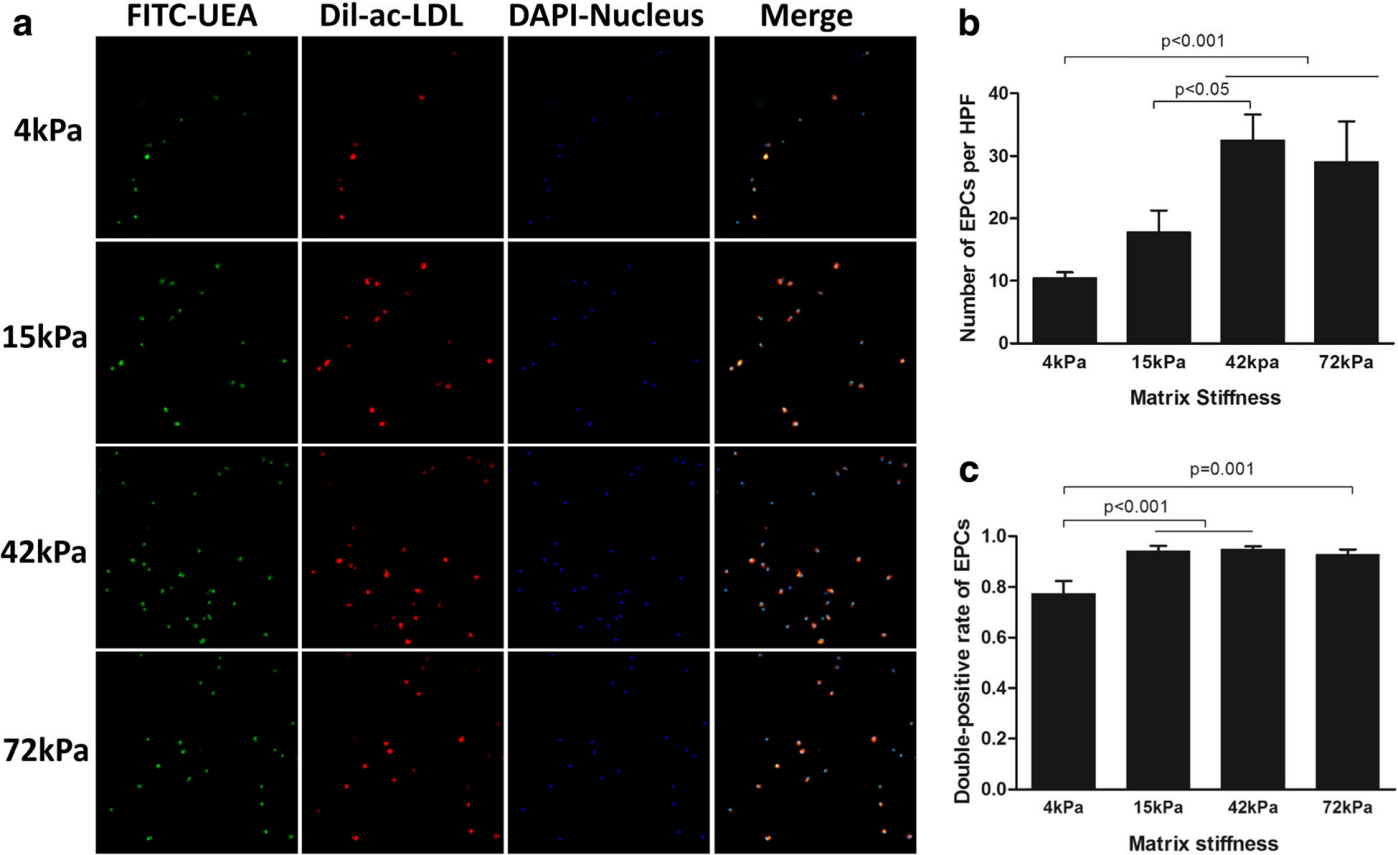
Double-positive cells for DiI-acLDL uptake and FITC-UEA-1 binding in CD34^–^ cell culture system with four different degrees of substrate stiffness. **a** Direct fluorescent staining; **b** comparisons of numbers of double-positive cells per HPF; **c** comparisons of rates of double-positive cells. Results are shown as mean + SD. *acLDL* acetylated low-density lipoprotein, *EPCs* endothelial progenitor cells, *HPF* high-power field, *acLDL* acetylated low-density lipoprotein, *DAPI* 4',6-diamidino-2-phenylindole, *FITC-UEA* fluorescein isothiocyanate-labeled *Ulex europaeus* agglutinin I lectin

Among mouse BMMNCs, the percentages of CD34^+^ and CD34^–^ cell subsets were 12.4% and 87.6%, respectively (Fig. 2a). After the purification with the MACS system, the purity of the CD34^+^ subset was ~ 95.0% (Fig. 2b). The numbers of CD34^+^ cells adherent to the different flexible substrates on day 7 after culture are presented in Fig. 3. Compared with those on compliant or soft culture substrates of 4 kPa and 15 kPa, the total number of CD34^+^ cells increased more when they were cultured on the moderately stiff substrate of 42 kPa (corresponding to the stiffness of myocardial ECM at days 7–14 postinfarction). In addition, the total number of CD34^+^ cells cultured on the 42-kPa substrate tended to increase more compared with the cells cultured on the 72-kPa stiff substrate (*P* = 0.058; Fig. 3a and b). Interestingly, the soft 4-kPa substrate significantly reduced the percentage of endothelial progenitor cells as evidenced by the alteration in DiI-acLDL and FITC-UEA-1 double-positive cells (*P* < 0.001; Fig. 3c). Meanwhile, the total number of CD34^–^ cells increased more when they were cultured on the moderately stiff 42-kPa substrate compared with the cells cultured on the soft substrates (4 kPa and 15 kPa; Fig. 4a and b). Again, the soft 4-kPa substrate significantly reduced the percentage of endothelial progenitor cells as evidenced by the alteration in DiI-acLDL and FITC-UEA-1 double-positive cells (*P* < 0.001; Fig. 4c). In addition, no significant differences in the percentage of DiI-acLDL/FITC-UEA-1 double-positive cells were observed among the 15-kPa, 42-kPa, and 72-kPa cell culture systems (*P* > 0.05; Figs. 3c and 4c). The overall impact of the flexible substrates on the CD34^+^ and CD34^–^ cell subsets was comparable, but the total number of CD34^–^ cells was significantly lower than that of CD34^+^ cells cultured under the same conditions (Figs. 3 and 4). In addition, the CD34^+^ cell culture system on the flexible substrates with the four different degrees of stiffness consistently displayed a much greater number of cells with endothelial progenitor characteristics than did the CD34^–^ culture system (*P* < 0.001).

### ECM stiffness regulated the expression of surface markers for endothelial cell lineage

**Fig. 5:**
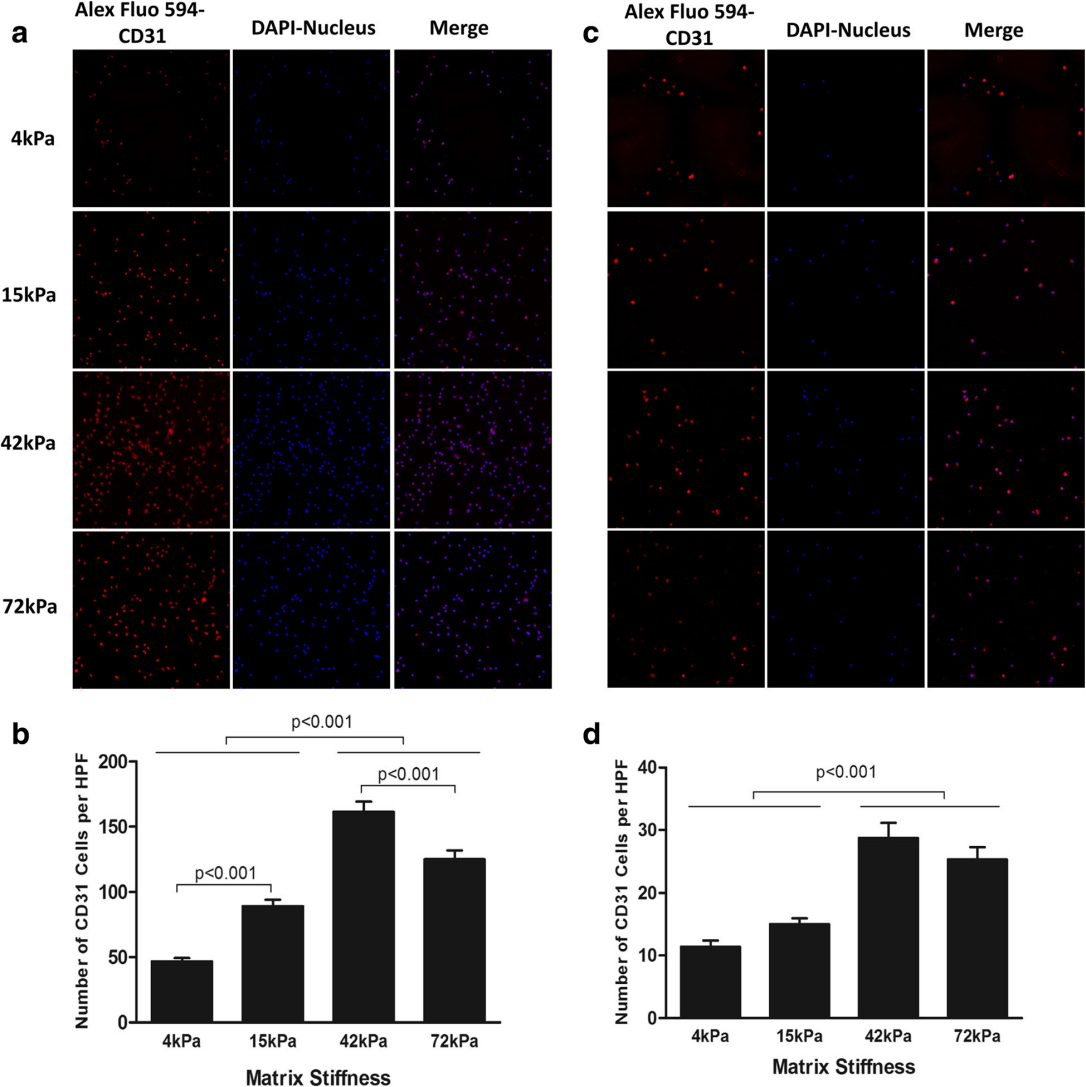
Endothelial lineage marker CD31 expression in CD34^+^ (**a**, **b**) or CD34^–^ (**c**, **d**) cell culture systems with four different degrees of substrate stiffness. **a**, **c** Direct fluorescent staining; **b**, **d** comparisons of numbers of double-positive cells per HPF. Results are shown as mean + SD. *DAPI* 4',6-diamidino-2-phenylindole, *HPF* high-power field

**Fig. 6:**
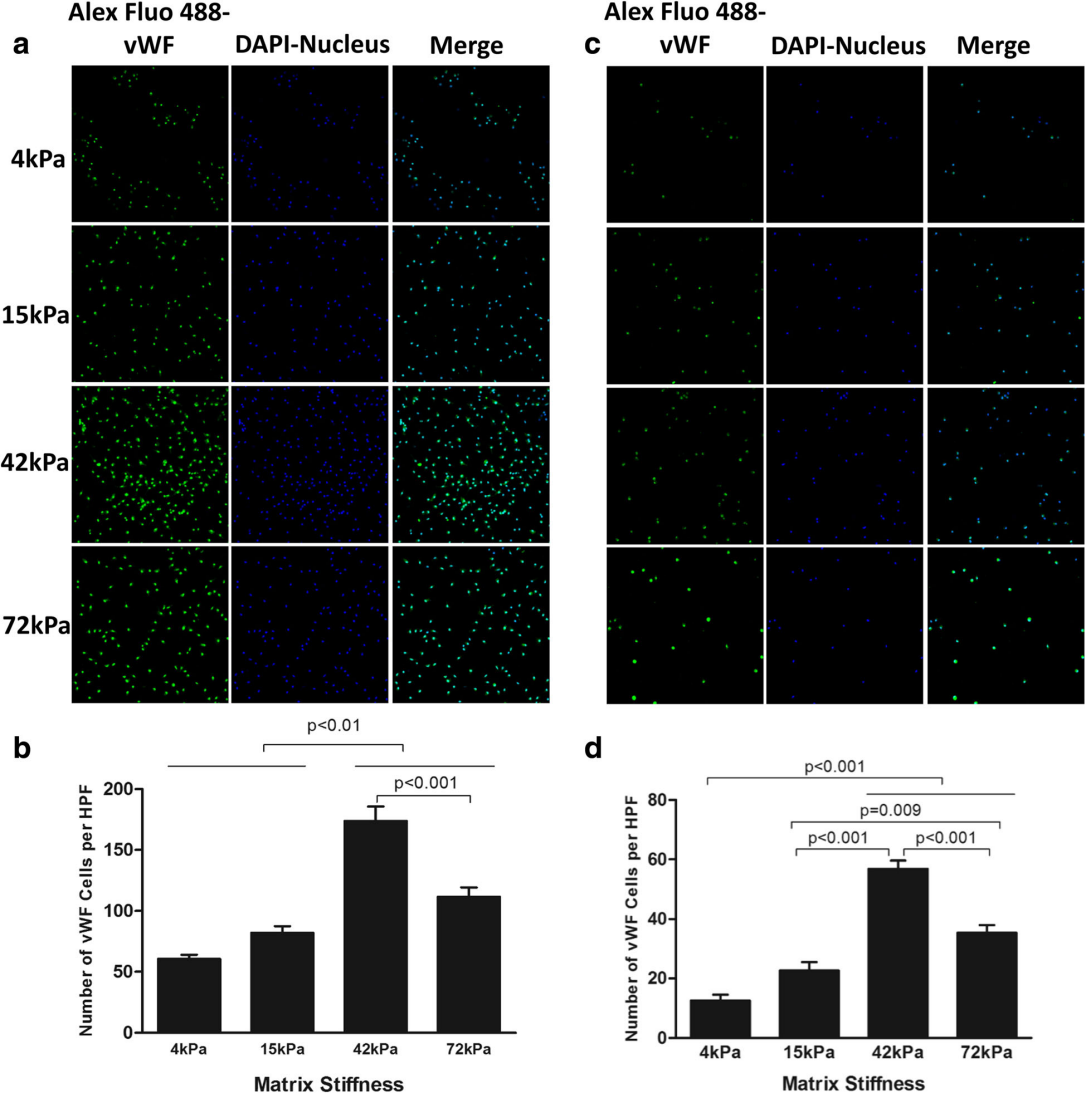
Endothelial lineage marker vWF expression in CD34^+^ (**a**, **b**) or CD34^–^ cells (**c**, **d**) cell culture system with four different degrees of substrate stiffness. **a**, **c** Direct fluorescent staining; **b**, **d** comparisons of numbers of double-positive cells per HPF. Results are shown as mean + SD. *DAPI* 4',6-diamidino-2-phenylindole, *HPF* high-power field, *vWF* von Willebrand factor

**Fig. 7:**
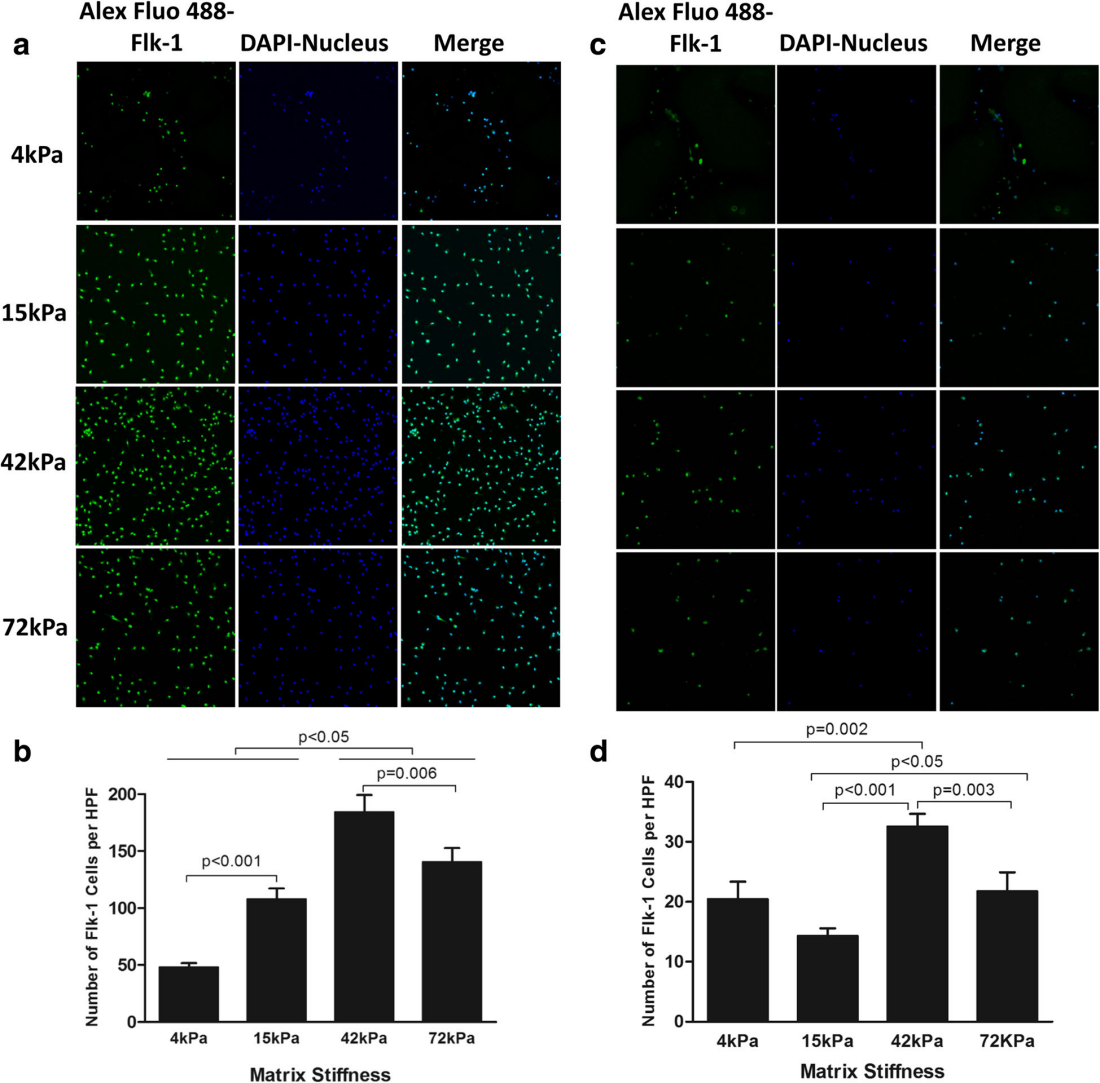
Endothelial lineage marker Flk-1 expression in CD34^+^ (**a**, **b**) or CD34^–^ cells (**c**, **d**) cell culture system with four different degrees of substrate stiffness. **a**, **c** Direct fluorescent staining; **b**, **d** comparisons of numbers of double-positive cells per HPF. Results are shown as mean + SD. *DAPI* 4',6-diamidino-2-phenylindole, *HPF* high-power field

**Fig. 8:**
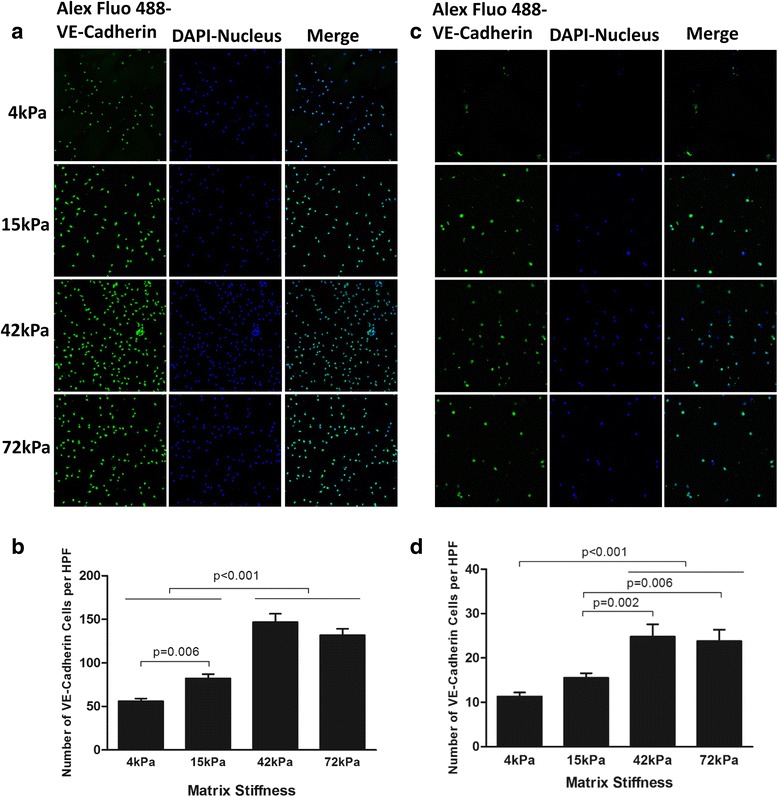
Endothelial lineage marker VE-Cadherin expression in CD34^+^ (**a**, **b**) or CD34^–^ cells (**c**, **d**) cell culture system with four different degrees of substrate stiffness. **a**, **c** Direct fluorescent staining; **b**, **d** comparisons of numbers of double-positive cells per HPF. Results are shown as mean + SD. *DAPI* 4',6-diamidino-2-phenylindole, *HPF* high-power field

The expressions of the vascular endothelial cell lineage markers CD31, vWF, Flk-1, and VE-cadherin were much greater in CD34^+^ cells compared with the CD34^–^ cells under all different culture conditions (Fig. 5a and c, Figs. 6 and 7, and Fig. 8a and c). Furthermore, the expression of these markers was consistently found to be the highest in CD34^+^ cells cultured on the moderately stiff 42-kPa substrate (*P* < 0.01; Fig. 5a and b, Figs. 6 and 7, and Fig. 8a and b). The expression of these markers was lowest in CD34^+^ cells cultured on the substrate with a stiffness of 4 kPa. Similarly, the expression of CD31, vWF, and VE-cadherin in CD34^–^ cells also gradually increased in the culture substrates from 4 kPa to 15 kPa to 72 kPa and to 42 kPa (Fig. 5c and d, Figs. 6 and 7, and Fig. 8c and d). These findings suggest that the moderately stiff substrate has an advantage over other culture substrates in the induction of endothelial cell lineage markers in both the CD34^+^ and CD34^–^ subsets.

### ECM stiffness regulates cytoskeleton formation and cytoskeleton arrangement in CD34^+^ cells

**Fig. 9:**
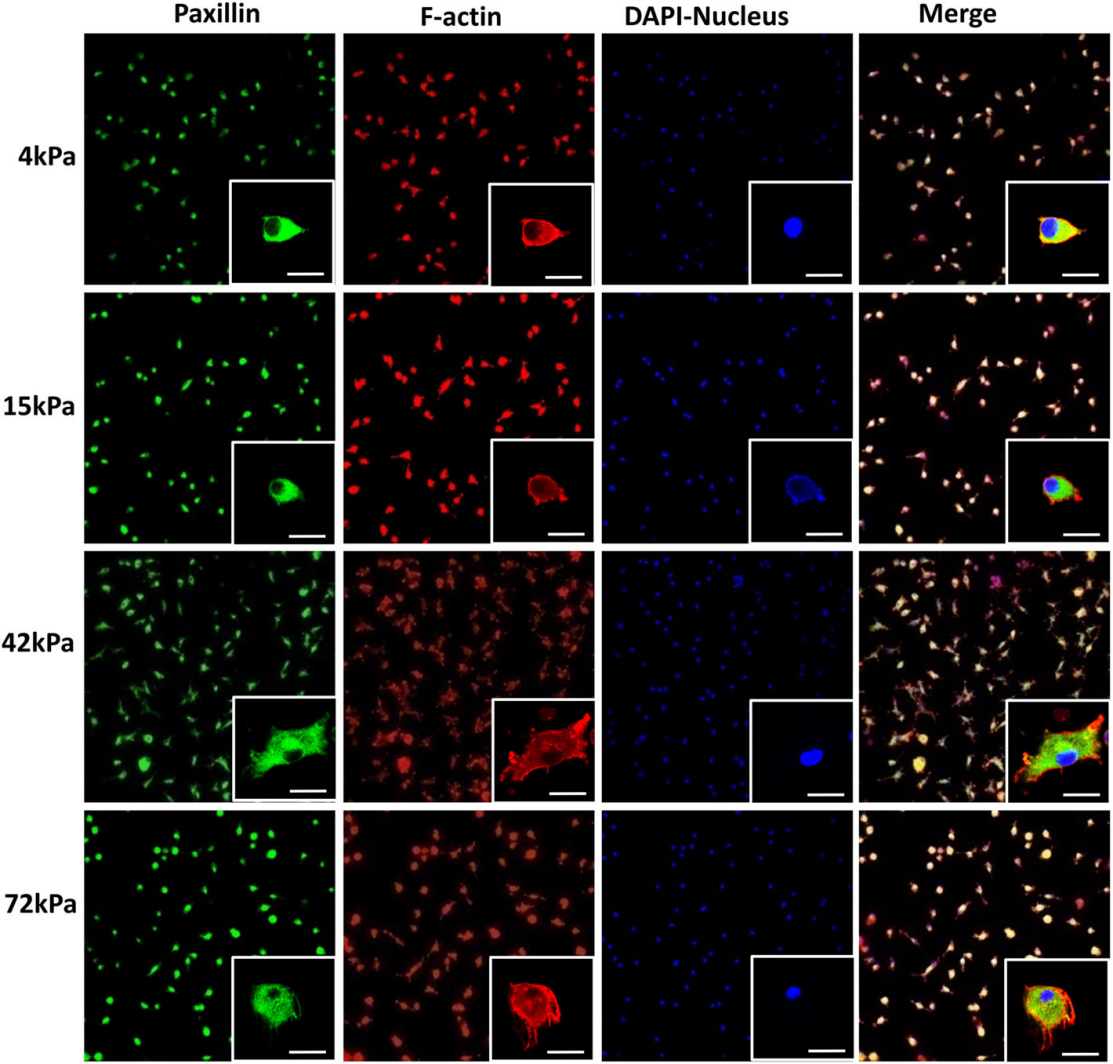
Cytoskeleton staining of CD34^+^ cells on the flexible substrates. The magnification of the objective lens of images in the lower right corners is 630×. *Scale bar* = 50 μm. *DAPI* 4',6-diamidino-2-phenylindole

**Fig. 10:**
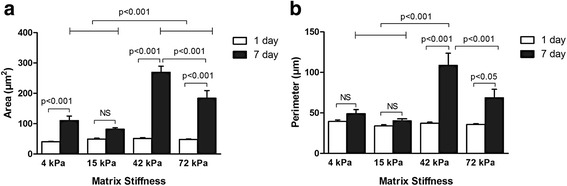
Comparisons of the surface areas (**a**) and circumferences (**b**) of adhesive cells among the flexible substrates. *NS* not significant

To confirm the cytoskeletal organization and focal adhesion, fluorescence localization of paxillin and F-actin of CD34^+^ cells on the flexible culture substrates was detected. The cells on the 4-kPa and 15-kPa substrates, the soft substrates, displayed a suborbicular shape with focal adhesions formed along the cell edge, and developed less lamellipodia (Fig. 9). Meanwhile, F-actin was highly enriched at the leading edge of the crawling cells. However, the F-actin network was organized indistinctly. In contrast, the adherent cells on the moderately stiff or stiff (42 kPa or 72 kPa) substrates, especially the 42-kPa substrate, were mostly elongated or spindle-like shaped with distinct perisomatic lamellipodia. Moreover, the F-actin network was circumferentially organized, increasingly at the leading edge of the adherent cells with increasing ECM stiffness (Fig. 9). Additionally, the surface areas and circumferences of cells on the moderately stiff and stiff (especially 42 kPa) substrates were dramatically more than those on the soft substrates at day 7 (Fig. 10a and b). The findings indicate that, compared with the soft substrates, the relatively rigid substrates, especially the 42-kPa substrate, prompted CD34^+^ cells to generate close contact with the ECM as well as to present a strong cell-spreading and lamellipodial protrusive activity.

### ECM stiffness regulatesd cell apoptosis and cell survival in CD34^+^ cells

**Fig. 11:**
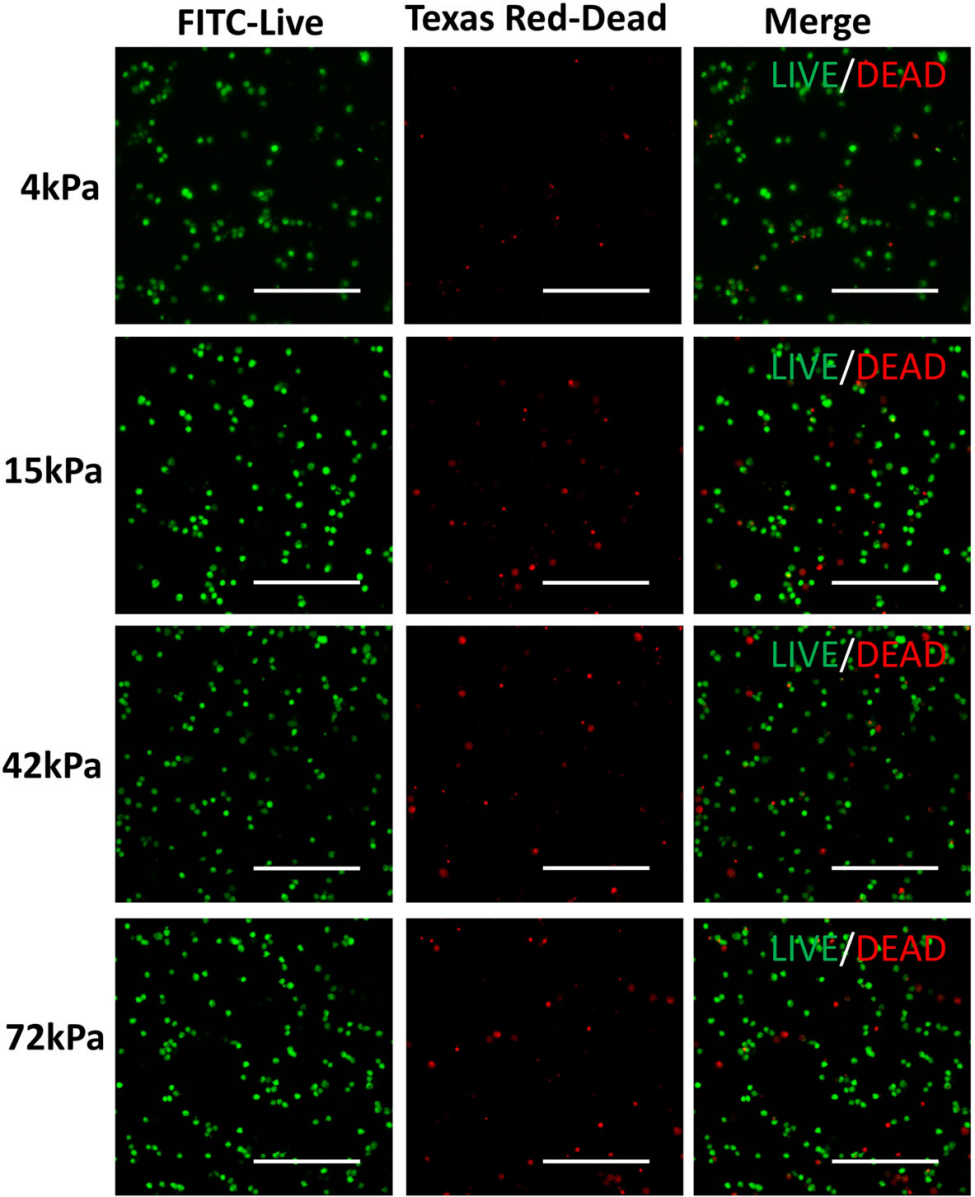
Live/Dead staining of CD34^+^ cells on the flexible substrates. *Scale bar* = 100 μm. *FITC* fluorescein isothiocyanate

**Fig. 12:**
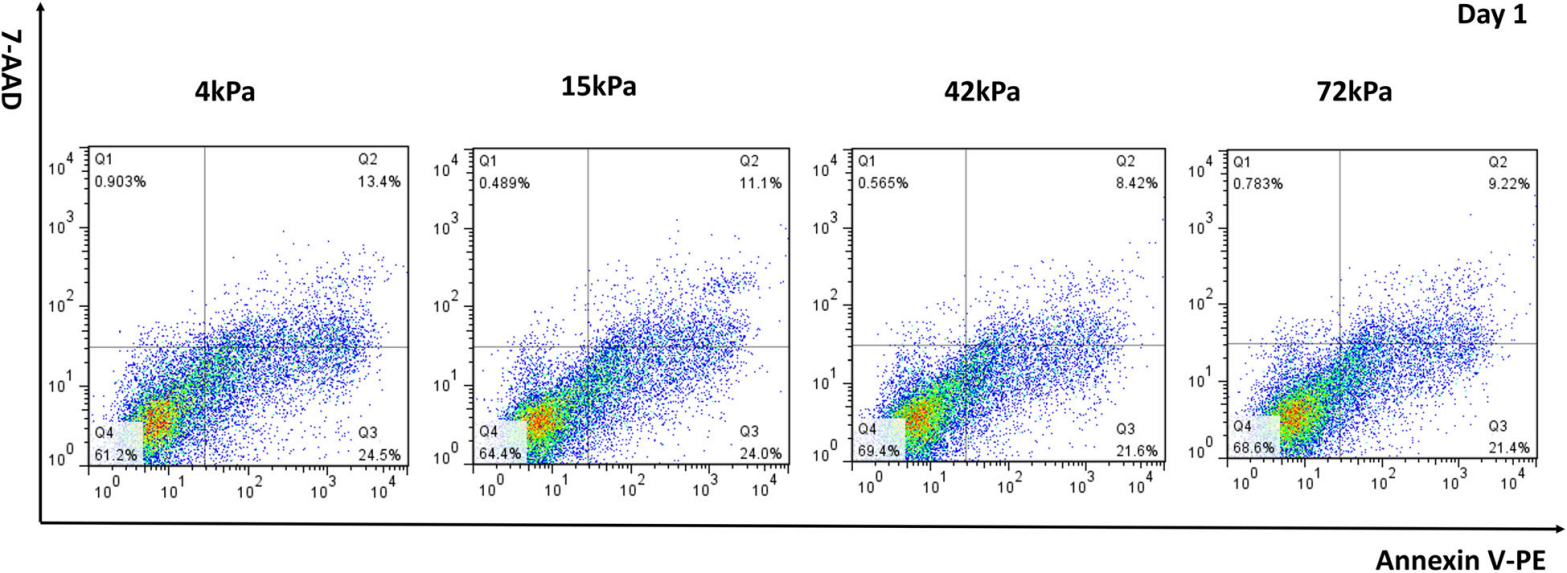
Flow cytometry for detection of consecutive apoptosis of CD34^+^ cells on the flexible substrates

To further understand the underlying mechanism of substrate stiffness on endothelial cell commitment and proliferation, we determined the percentage of apoptosis of CD34^+^ cells cultured on the flexible substrates using Live Green vial/Dead Red vial co-staining assay. The percentage of dead cells on the soft substrates (4 kPa or 15 kPa) was more than that on the moderately stiff or stiff substrates (42 kPa or 72 kPa) (Fig. 11). Furthermore, CD34^+^ cells were also co-stained with Annexin V and 7-AAD to detect early or late apoptosis by flow cytometry. The proportion of early cell apoptosis on the 4-kPa, 15-kPa, 42- kPa, and 72-kPa substrates was 24.5%, 24.0%, 21.6%, and 21.4%, respectively. The rate of late cell apoptosis was 13.4%, 11.1%, 8.4%, and 9.2%, respectively (Fig. 12). Thus, CD34^+^ cells on the soft substrates had higher percentages of apoptosis compared with the cells cultured on the moderately stiff or stiff substrates. These findings might partially explain the advantage of the relatively rigid substrates (especially at 42 kPa) in promoting CD34^+^ cell survival.

## Discussion

In the present study, we have provided mechanistic insights into the impacts of infarcted myocardial ECM stiffness on the endothelial cell lineage commitment of bone marrow-derived CD34^+^ and CD34^–^ cell subsets. Our results demonstrate that infarcted myocardium-like ECM stiffness regulates the cytoskeletal arrangement, cell survival, cell-ECM adhesion, and cell differentiation in both CD34^+^ cells and CD34^–^ cells derived from BMMNCs. Moreover, there were significant differences in the specification of the endothelial cell lineage between the two cell subsets induced on the flexible culture substrates, which were distinct from conventional glass rigid substrates. The matrix stiffness of 42 kPa, corresponding to myocardial ECM at days 7–14 after MI, was more suitable for the induction of FITC-UEA-1 and DiI-acLDL double-positive cells, as well as the expression of endothelial cell lineage markers such as CD31, vWF, Flk-1, and VE-cadherin in both CD34^+^ and CD34^–^ subsets. Meanwhile, in the cell culture system with the flexible substrates, the CD34^+^ cell subset showed higher endothelial lineage commitment compared with the CD34^–^ subset under various culture conditions. Thus, it is clear that an optimal ECM stiffness promotes the endothelial lineage commitment of bone marrow-derived CD34^+^ cells in vitro. The combination of an optimal cell subset and a suitable ECM stiffness may provide a potentially useful strategy to enhance cell-based cardiac repair after MI.

For the repair of ischemia or infarcted myocardium, the addition of the proper stem/progenitor cells to the suitable microenvironment seems to be promising via promoting neovascularization [14–16]. The definition and classification of stem/progenitor cells mainly depends on the cell surface antigens. CD34 is an important cell surface marker of hematopoietic progenitor cells. Bone marrow-derived CD34^+^ cells have the potent potential to differentiate into endothelial lineage cells which are deeply involved in neovascularization [17]. Meanwhile, the phenotype CD34 functions to mediate the attachment of cells to the ECM [18]. In the present study, we found significant differences in cell attachment to the flexible substrates between the CD34^+^ cell subset and the CD34^–^ subset. Furthermore, as compared with the CD34^–^ subset, the CD34^+^ subset was more easily induced into the endothelial cell lineage, which potentially facilitates angiogenesis as well as cardiac repair. The difference in specification efficiency between the two cell subsets might result from the differences in cell attachment to the flexible substrates. Furthermore, in terms of the CD34^+^ cell subset, the present study shows a significant difference in the number of cells expressing endothelial phenotypes (not the percentage) on the flexible substrates. However, there exists a consistently high percentage of cells expressing endothelial phenotypes on the 15- to 72-kPa substrates. This suggests that the significant differences in endothelial phenotype expression might result from differences in the cell adherence capacity. In the present study, CD34^+^ cells seemed to more easily adhere to the flexible substrates than the CD34^–^ cells. Mechanically, the characteristics of the CD34 antigen in improving cell adherence to the ECM might provide an explanation for the preference of CD34^+^ cell survival and specification on the flexible substrates. In contrast, CD34^–^ cells presented a lower survival and specification ratio. In addition, CD34^+^ cells showed distinct focal adhesion, cytoskeletal organization, and cellular morphology on the flexible substrates with varied stiffness. Moreover, cytoskeletal architecture and cell-ECM adhesions become increasingly organized with increasing stiffness. Paxillin, as a connection between ECM and cells, regulates cell fate and even cell specification by influencing cell attachment and cytoskeleton formation (mainly referring to the F-actin network) [19]. Cell adhesion and apoptosis were both regulated by cell-ECM interaction. Disruption of the cell-ECM interaction promotes cell apoptosis [20]. In the present study, CD34^+^ cells had a lower apoptotic rate on the moderately stiff or stiff substrates (42 kPa or 72 kPa) than those on the soft substrates (4 kPa and 15 kPa). Based on the analysis on the cytoskeleton and cell morphology, the differences in cell apoptosis might relate to the higher adhesive strength of CD34^+^ cells to the relatively rigid substrates or the stronger cell-ECM interaction.

On the other hand, cell-ECM interaction is also widely believed to play an important role in cell survival and differentiation [21]. Stem/progenitor cells are able to sense and respond to the surrounding tissue physical microenvironment, which is known as the ECM stiffness [22]. Accumulating data have shown that tissue ECM stiffness plays an important role in stem cell adhesion, survival, and lineage commitment [23, 24]. Following MI, myocardial ECM stiffness might be an important physical condition impacting the efficacy of cell implantation by influencing these cellular biological behaviors [8, 9, 25]. Thus, the simulation in vitro to the myocardial physical microenvironment post-MI is thought to be essential in detecting implanted cell biology and validating an optimal cell therapy strategy. Indeed, in the present study, infarcted myocardium-like ECM stiffness showed a significant influence on the potential pro-angiogenesis ability of bone marrow-derived CD34^+^ cells. Furthermore, myocardial ECM at days 7–14 post-MI might offer an optimal physical microenvironment for commitments of engrafted pluripotent cells to endothelial cell lineage, which suggests that the beneficial effect on infarcted myocardium repair might be time- or stiffness-dependent. Moreover, CD34^+^ cells under induction of ECM stiffness present a similar stiffness-dependent differentiation principle as BMMNCs, and might consequently exert an important role in regulating the efficacy of cell implantation for the damaged myocardium, as reported in our previous study [9]. Furthermore, these findings might partially explain the optimal timing of stem cell implantation after MI.

Notably, almost all of the previously published randomized controlled trials (RCTs) consistently performed cell therapy at days 0 to 7 after MI [26]. Moreover, our previous meta-analysis on these RCTs indicated the more favorable effect of bone marrow-derived stem cell engraftment at 4–7 days after MI on improving left ventricular ejection farction (LVEF) and decreasing left ventricular (LV) end-systolic dimensions than a procedure performed within 24 h following MI [27]. Since days 7–14 post-MI is a “time-domain blank zone” in previous clinical studies on cell-based cardiac repair, it may be important to further investigate the biological behavior of engrafted cells and the efficacy of cell therapy within this “time-domain blank zone” due to the unavoidable therapeutic delay for acute MI. Our previous studies verified the optimal efficacy of cell therapy at 7–14 days after MI, which might relate to ECM stiffness-dependent angiogenesis [9]. Overall, the procedure of cell therapy either too early or too late after acute MI was not productive in terms of promoting cardiac repair due to the absence of the “suitable” stiffness of myocardial ECM. Although the previous clinical trials confirmed the efficacy of bone marrow-derived cell implantation within 24 h or more than 30 days after MI [28, 29], the magnitudes of the beneficial effects were significantly different. Based on our present findings, the time-dependent changes in myocardial ECM stiffness after MI may contribute to the differing efficacy of cell therapy at these different time points.

The limitations of our study deserve comment. Firstly, the adhesion ability of cells on the flexible (4–72 kPa) substrates was relatively low. The less cell-cell contact might influence cell proliferation. Thus, we did not further investigate the impact of the flexible substrates on cell proliferation in this study. Secondly, due to insufficient cell availability and restricted conditions of the special culture system, the impact of substrate stiffness on cell migration was not elucidated. Additionally, the findings in the present study were achieved in a two-dimensional in vitro model, and needs to be verified under three-dimensional conditions in vivo.

## Conclusions

Here, we demonstrated that infarcted myocardium-like ECM regulated the endothelial lineage commitment of bone marrow-derived CD34^+^ and CD34^–^ cells. Bone marrow-derived CD34^+^ cells showed greater adherence and survival ability than CD34^–^ cells on the flexible substrates. Moreover, the CD34^+^ cell subset was prone to effective specification into endothelial lineage cells under induction on a matrix with a stiffness of 42 kPa (corresponding to myocardial ECM stiffness at 7–14 days post-infarction) than with other matrix stiffnesses. Overall, the combination of a bone marrow-derived CD34^+^ cell subset with ECM of an optimal stiffness might be a potential solution to promoting angiogenesis or neovasculogenesis within damaged myocardium following MI. The present study provides us with an insight into the optimized therapeutic strategy for cell-based cardiac repair in experimental animals.

## Additional file

Additional file 1: Immunofluorescent staining of Fibronectin coated on the four flexible substrates. (JPG 5960 kb)

## Abbreviations

acLDL: Acetylated low-density lipoprotein
BMMNC: Bone marrow-derived mononuclear cell
BSA: Bovine serum albumin
DAPI: 4',6-diamidino-2-phenylindole
ECM: Extracellular matrix
FBS: Fetal bovine serum
FITC-UEA-1: Fluorescein isothiocyanate-*Ulex europaeus* agglutinin-1
HPF: High-power field
MACS: Magnetic activated cell sorting
MI: Myocardial infarction
PA: Polyacrylamide
PBS: Phosphate-buffered saline
RCT: Randomized controlled trial
vWF: Von WIllebrand factor
