# Supplementary figures and images for: Bone marrow CD34+ cell subset under induction of moderate stiffness of extracellular matrix after myocardial infarction facilitated endothelial lineage commitment in vitro
Source: Stem Cell Res Ther. 2017 Dec 13;8:280. doi: 10.1186/s13287-017-0732-x (PMC5729449; doi:10.1186/s13287-017-0732-x)

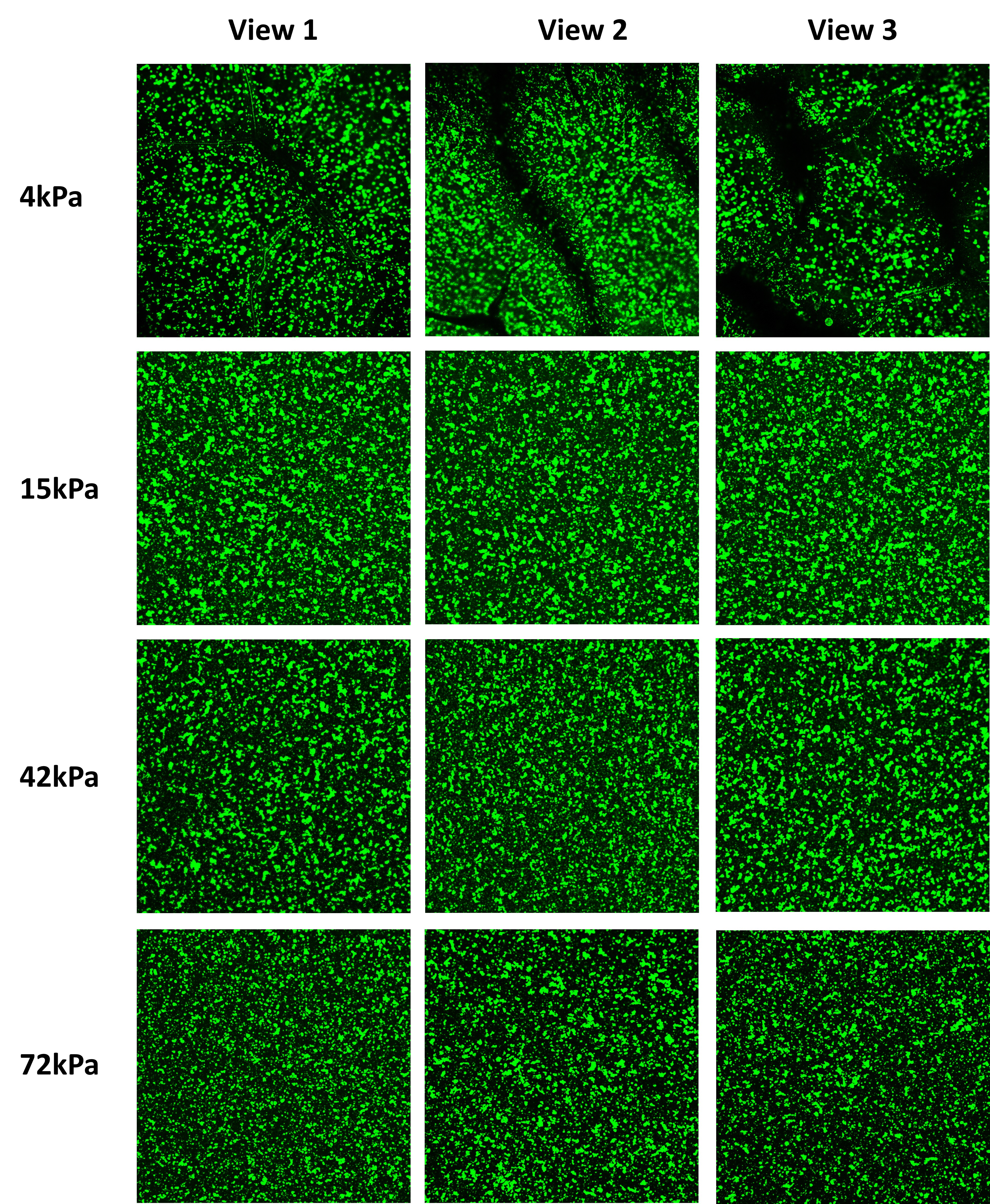

Supplement: Additional file 1: — Immunofluorescent staining of Fibronectin coated on the four flexible substrates. (JPG 5960 kb) [file 13287_2017_732_MOESM1_ESM.jpg]
